# Virome Status of Preclonal Candidates of Grapevine Varieties (*Vitis vinifera* L.) From the Slovenian Wine-Growing Region Primorska as Determined by High-Throughput Sequencing

**DOI:** 10.3389/fmicb.2022.830866

**Published:** 2022-02-21

**Authors:** Vanja Miljanić, Jernej Jakše, Urban Kunej, Denis Rusjan, Andreja Škvarč, Nataša Štajner

**Affiliations:** ^1^Department of Agronomy, Biotechnical Faculty, University of Ljubljana, Ljubljana, Slovenia; ^2^Chamber of Agriculture and Forestry of Slovenia, Agriculture and Forestry Institute Nova Gorica, Nova Gorica, Slovenia

**Keywords:** *Vitis vinifera* L., preclonal candidates, HTS, viruses, viroids

## Abstract

Diseases caused by viruses and virus-like organisms are one of the major problems in viticulture and grapevine marketing worldwide. Therefore, rapid and accurate diagnosis and identification is crucial. In this study, we used HTS of virus- and viroid-derived small RNAs to determine the virome status of Slovenian preclonal candidates of autochthonous and local grapevine varieties (*Vitis vinifera* L.). The method applied to the studied vines revealed the presence of nine viruses and two viroids. All viral entities were validated and more than 160 Sanger sequences were generated and deposited in NCBI. In addition, a complete description into the co-infections in each plant studied was obtained. No vine was found to be virus- and viroid-free, and no vine was found to be infected with only one virus or viroid, while the highest number of viral entities in a plant was eight.

## Introduction

Grapevine is one of the most important fruit crops by acreage and economic importance (Torregrosa et al., 2015). According to the International Organization of Vine and Wine Intergovernmental Organization (OIV), 7.4 million hectares around the world were planted with grapevines in 2018, and vineyards in Spain, China, France, Italy, and Turkey represented 50% of the total world cultivated grapevine area. According to the OIV, the world production of grapes in 2018 was 77.8 million tons. In Slovenia in 2018, grapevines occupied an area of 15,630 hectares and annual production of grapes was 126,958 tons.^1^

Grapevine may harbor more than 86 viruses and viroids, belonging to different families and genera (Fuchs, 2020). Viruses and virus-like organisms cause severe damage to grapevine production worldwide. They cause leaf degeneration, malformation, puckering, leaf rolling, chlorosis, necrosis, ringspots, line patterns, mosaic patterns, vein-banding, vein-clearing, stunting, wilting, shortened internodes, fasciation, zigzag growth, grooving, cracking, and pitting of wood (Credi et al., 1981; Mavrič et al., 2003; Andret-Link et al., 2004; Bouyahia et al., 2005; Al Rwahnih et al., 2009; Lunden et al., 2010; Giampetruzzi et al., 2012; Mavrič Pleško et al., 2014; Sudarshana et al., 2015). They impact vine yield and wine quality, as viral entities delay ripening, affect grape quality, decrease sugar content, affect the content of pigments, various aromatic components and other metabolites, and increase the acidity of wines (Lee and Martin, 2009; Vega et al., 2011; Alabi et al., 2016; Girardello et al., 2020; Lee et al., 2021). Viral entities eventually lead to the death of chronically infected plants.

Therefore, rapid and accurate diagnosis and identification is very important. Most methods for detection and identification require prior knowledge of the potential pathogens (e.g., use of antibodies in serological methods or virus specific primers in PCR amplification), with the exception of the metagenomic approach called high-throughput sequencing technology (HTS). HTS is a powerful technology that enables rapid detection of viral entities in plant tissues, including unknown as well as known viruses and viroids in symptomatic and asymptomatic plants, without the need for prior knowledge (Al Rwahnih et al., 2009; Kreuze et al., 2009; Fajardo et al., 2020). HTS of small RNAs (small RNA sequencing; sRNA-seq) has been shown to be efficient in detecting plant viruses or viroids (Kreuze et al., 2009; Kashif et al., 2012; Vives et al., 2013; Jakse et al., 2015; Singh et al., 2020). This approach exploits a natural antiviral defense mechanism called RNA silencing or RNA interference (RNAi). The silencing mechanism is initiated by RNase III-like enzymes called Dicer-like enzymes (DCL) which cleave long double-stranded RNAs (dsRNAs) into short interfering (si)RNA and miRNA precursors with a hairpin or stem-loop structure into miRNA duplexes (miRNA/miRNA*) (Bernstein et al., 2001; Bartel, 2004; Baulcombe, 2004). During the process of viral infection small RNAs (sRNAs) accumulate abundantly in plants and can be detected by deep sequencing of infected plants. sRNA-seq provides a unique opportunity to easily detect and identify grapevine viruses and viroids due to the abundance of sRNAs (Navarro et al., 2009; Giampetruzzi et al., 2012; Eichmeier et al., 2016; Czotter et al., 2018; Demian et al., 2020; Li et al., 2021).

Slovenia is a traditional wine-growing country with many local and indigenous grapevine varieties revitalized in current clonal selection programs, according to the rules on the marketing of material for the vegetative propagation of vine (Official gazette, N°93/05 and 101/20) and OIV process for the clonal selection of vines (Resolution oiv-viti-564a-2017). In the past, propagation material was controlled just visually, which led to uncontrolled spread of viruses.

The aim of the presented work was to investigate the virome status of Slovenian preclonal grapevine candidates and to study their genetic diversity and co-infections using identification by sRNA-seq and confirmation by RT-PCR and Sanger sequencing.

## Materials and Methods

### Plant Material

A total of 82 dormant cuttings of 6 preclonal grapevine varieties (*Vitis vinifera* L.)—2 reds, “Refošk” (“Terrano”) and “Pokalca” (“Schioppettino”), and 4 whites, “Laški rizling” (“Welschriesling”), “Rebula” (“Ribolla Gialla”), “Malvazija” (“Malvasia d’Istria”), and “Zeleni Sauvignon” (“Sauvignon Vert”), were collected from the 3 vineyards [Pouzelce (P); Base (B) and Genebank (G), referenced to Table 1)] maintained by Centre of grapevine selection (STS Vrhpolje, Vipava) in Primorska wine-growing region in Slovenia in February 2019. After 3–4 weeks in water at room temperature one-bud cuttings started bud-bursting and the obtained leaves were collected and stored at –80°C for further analysis.

**TABLE 1:** Viruses and viroids detected in 12 libraries using the VirusDetect approach.

Library labels	Samples	Detected viruses and viroids	Reference sequence	Reference length	Consensus length	Reference coverage (%)	No. of contigs	Sequencing depth
005	Laški rizling 3/34B	RBDV (RNA1)	AB948214	5,449	5,449	100	2	1556.2
	Laški rizling 3/45B	RBDV (RNA2)	AB948215	2,231	2,202	98.7	4	513.8
	Laški rizling 3/56B	GPGV	KP693444	7,172	6,957	97	8	487.4
	Laški rizling 3/64B	GRSPaV	AY881627	8,743	7,434	85	66	9.8
		GFkV	AJ309022	7,564	6,210	82.1	55	49.3
		GRVFV	KY513701	6,730	2,669	39.7	55	15.5
		GSyV-1	KP221269	334	150	44.9	3	20.9
		HSVd	KJ810551	309	309	100	3	2223.0
		GYSVd-1	AB028466	368	368	100	5	971.9
006	Refošk 9/3B	GPGV	FR877530	7,259	7,257	100	7	123.3
	Refošk 10/1B	GRSPaV	KX035004	8,743	8,666	99.1	41	16.6
	Refošk 10/2B	GRGV	KX171166	6,863	4,362	63.6	40	53.0
	Refošk 10/3B	GRVFV	KY513702	6,716	3,420	50.9	34	46.3
		HSVd	KJ810551	309	309	100	2	1856.0
		GYSVd-1	KP010010	389	389	100	3	1505.9
007	Rebula 15/1B	GPGV	FR877530	7,259	7,248	99.8	8	53.5
	Rebula 15/2B	GFkV	KT000362	7,564	5,814	76.9	60	13.2
	Rebula 15/3B	GRVFV	KY513702	6,716	2,483	37	40	14.6
	Rebula 16/1B	HSVd	KJ810551	309	309	100	3	490.5
	Rebula 16/2B	GYSVd-1	KP010010	389	389	100	3	374.2
	Rebula 16/3B							
	Rebula 19/1B							
	Rebula 19/2B							
008	Rebula 19/3B	GPGV	KY747494	7,156	7,135	99.7	9	56.5
	Rebula 20/3B	GRSPaV	KR054734	8,753	7,168	81.9	70	5.6
	Rebula 22/1B	GFkV	AJ309022	7,564	5,815	76.9	46	29.0
	Rebula 22/2B	GRVFV	KY513702	6,716	5,706	85	55	93.3
	Rebula 22/3B	HSVd	KY508372	316	314	99.4	3	650.5
	Rebula 24/2B	GYSVd-1	AB028466	368	368	100	6	298.8
	Rebula 26/1B							
	Rebula 26/2B							
	Rebula 26/3B							
009	Malvazija 32/1B	GPGV	FR877530	7,259	7,259	100	5	142.5
	Malvazija 32/2B	GRSPaV	KX035004	8,743	7,838	89.6	70	7.0
	Malvazija 32/3B	GSyV-1	KP221256	6,482	3,160	48.8	25	29.4
	Malvazija 32/9B	HSVd	KJ810551	309	309	100	4	1462.5
		GYSVd-1	KJ466324	367	367	100	5	2201.1
010	Refošk 9/3P	GPGV	FR877530	7,259	7,248	99.8	16	93.5
	Refošk 9/4P	GRSPaV	KX035004	8,743	7,595	86.9	76	6.5
	Refošk 9/5P	GLRaV-3	GQ352631	18,498	18,234	98.6	23	39.6
	Refošk 10/2P	GFkV	AJ309022	7,564	6,137	81.1	52	69.5
	Refošk 10/3P	GRGV	KX109927	6,863	3,144	45.8	36	22.9
	Refošk 10/5P	GRVFV	KY513702	6,716	4,752	70.8	90	33.1
	Refošk 11/2P	HSVd	KJ810551	309	309	100	5	889.0
	Refošk 11/3P	GYSVd-1	KP010010	389	389	100	4	694.1
	Refošk 11/4P							
011	Refošk 12/1P	GPGV	FR877530	7,259	7,246	99.8	6	83.2
	Refošk 12/3P	GRSPaV	KX274274	8,725	7,663	87.8	65	6.3
	Refošk 12/6P	GFkV	KF417610	532	177	33.3	3	6.8
	Refošk 12/18P	GRVFV	KY513701	6,730	1,852	27.5	30	5.4
	Refošk 12/19P	HSVd	KJ810551	309	309	100	3	503.7
	Refošk 12/20P	GYSVd-1	KP010010	389	389	100	2	565.2
012	Zeleni Sauvignon 14/2P	GPGV	FR877530	7,259	7,240	99.7	11	119.6
	Zeleni Sauvignon 14/5P	GRSPaV	KT008379	780	486	62.3	8	6.7
	Zeleni Sauvignon 14/6P	GFkV	AJ309022	7,564	5,984	79.1	54	28.5
	Zeleni Sauvignon 14/7P	GRVFV	KY513701	6,730	2,927	43.5	47	9.0
	Zeleni Sauvignon 15/1P	HSVd	KJ810551	309	309	100	3	279.5
	Zeleni Sauvignon 15/2P	GYSVd-1	KP010010	389	389	100	2	452.0
	Zeleni Sauvignon 15/3P							
	Zeleni Sauvignon 16/1P							
	Zeleni Sauvignon 16/2P							
013	Zeleni Sauvignon 16/3P	GPGV	KY747494	7,156	7,135	99.7	14	122.9
	Zeleni Sauvignon 24/3P	GRSPaV	MG938309	8,753	4,240	48.4	63	9.5
	Zeleni Sauvignon 24/9P	GFLV (RNA1)	JX513889	7,340	6,752	92	31	149.6
	Zeleni Sauvignon 24/10P	GFLV (RNA2)	JX559643	3,769	3,417	90.7	12	214.9
	Zeleni Sauvignon 24/11P	GRVFV	MH544692	494	149	30.2	3	5.6
	Zeleni Sauvignon 26/1P	HSVd	KJ810551	309	309	100	3	412.4
	Zeleni Sauvignon 26/2P	GYSVd-1	AB028466	368	368	100	6	97.4
	Zeleni Sauvignon 26/3P							
014	Malvazija 20/2P	GPGV	FR877530	7,259	6,917	95.3	38	10.7
	Malvazija 20/6P	GFkV	AJ309022	7,564	2,302	30.4	41	5.3
	Malvazija 20/7P	GRVFV	KY513702	6,716	3,227	48	64	11.0
	Malvazija 20/46P	HSVd	KY508372	316	314	99.4	2	232.1
	Malvazija 20/47P	GYSVd-1	KP010010	389	389	100	2	394.9
	Malvazija 20/48P							
	Malvazija 20/50P							
015	Malvazija 21/6P	GPGV	FR877530	7,259	7,247	99.8	12	33.2
	Malvazija 21/7P	GRSPaV	FJ943281	780	635	81.4	9	7.3
	Malvazija 21/8P	GFkV	AJ309022	7,564	2,432	32.2	36	6.4
	Malvazija 21/20P	GRVFV	KY513701	6,730	4,364	64.8	94	20.7
	Malvazija 23/2P	HSVd	KJ810551	309	309	100	5	473.3
	Malvazija 23/3P	GYSVd-1	AB028466	368	368	100	5	394.1
	Malvazija 23/4P							
016	Pokalca 3/4P	GPGV	FR877530	7,259	7,243	99.8	12	40.7
	Pokalca 3/5P	GRSPaV	KX958435	8,743	5,728	65.5	86	5.3
	Pokalca 3/6P	GFLV (RNA1)	KX034843	7,347	5,268	71.7	21	737.3
	Pokalca 9/2G	GFLV (RNA2)	GQ332370	3,773	3,475	92.1	18	752.5
	Pokalca 9/3G	GRVFV	KY513702	6,716	4,627	68.9	84	28.9
	Pokalca 9/26G	HSVd	KJ810551	309	309	100	2	371.0
	Pokalca 9/27G	GYSVd-1	AB028466	368	368	100	4	116.2

### High-Throughput Sequencing of Virus- and Viroid-Derived Small RNAs

Eighty-two grapevine samples were pooled into 12 bulks/libraries, each bulk representing samples of the same variety; some varieties were represented with more than one bulk (Table 1). sRNAs were extracted by an enrichment procedure using a mirVana miRNA Isolation Kit (Ambion, Life Technologies) according to the manufacturer’s instructions. sRNA libraries were prepared using the Ion Total RNA-Seq kit and checked for quality using an Agilent 2100 Bioanalyzer (Agilent Technologies). Barcode-labeled cDNA libraries were sequenced on an Ion PI chip v3 using an Ion Proton Sequencer (Ion Torrent; Life Technologies) according to the manufacturer’s instructions. According to the Ion Torrent sequencing pipeline, raw reads had removed adapter sequences and they were deposited in the National Center for Biotechnology Information (NCBI) Sequence Read Archive (SRA) database under BioProject accession number PRJNA765925.

### Analysis of High-Throughput Sequencing Data

Raw sequence reads were filtered based on quality score and read length using cutadapt tools (Martin, 2011). VirusDetect^2^ (Zheng et al., 2017), an automated bioinformatics pipeline, was employed for further analysis of obtained sequences. VirusDetect is a highly sensitive and enables efficient analysis of sRNA datasets for viral identification. The software package first align sRNA reads to viral GenBank references using Burrows-Wheeler Aligner (BWA). The mapped sRNA reads are then assembled into contigs according to the viral reference. This program also maps the sRNA reads to host reference sequences to discard host-derived sRNAs. VirusDetect performs *de novo* assembly of sRNAs using Velvet with automated parameter optimization. Contigs obtained from *de novo* assemblies were aligned to the grapevine genome and all contigs with nucleotide identity greater than 90% with the grapevine genome were discarded. *De novo* assembled contigs were concatenated with reference-guided generated contigs and then all redundancies were removed, according to the employed iAssembler pipeline. The obtained contigs were then compared with the viral GenBank references for their identification. This automated pipeline used the BLASTN algorithm to compare the contigs to the reference virus nucleotide sequences and BLASTX algorithm to compare the contigs to the reference virus protein sequences (Zheng et al., 2017).

### Validation of Predicted Infections by RT-PCR, Direct Sanger Sequencing and Cloning, Sequence Analysis and Phylogenetic Studies

Validation of HTS results of predicted infections was performed by RT-PCR and Sanger sequencing. Total RNAs for all individual samples were extracted from 70 to 100 mg of frozen leaves using Monarch RNA Total Miniprep Kit (New England Biolabs) following recommended instructions. The RNAs concentration and purity were assessed with NanoVue Plus spectrophotometer (GE Healthcare Life Sciences). Due to the low RNA concentration and purity, three samples (Malvazija 20/2P, Malvazija 21/7P and Malvazija 23/4P) were excluded from further analysis. RT−PCRs were performed using a two−step protocol, where total RNAs were first reverse transcribed using High-Capacity cDNA Reverse Transcription Kit (Applied Biosystems) according to the manufacturer’s instructions followed by PCR with specific primers (Supplementary Table 2). The PCR reaction mixture (20 μL total) contained 10.7 μL nuclease-free water, 4 μL 5 × PCR buffer (Promega), 1.6 μL MgCl_2_ (Kapa Biosystems), 1.6 μL dNTP mix (10 mM each of the 4 dNTPs) (Promega), 0.5 μL of each primer, 0.1 μL KAPA Taq DNA polymerase (Kapa Biosystems), and 1 μL of cDNA. RT-PCR products were analyzed by electrophoresis on a 1.4% agarose gel, stained with ethidium bromide, and visualized under a UV transilluminator and remaining reaction was cleaned by Exo-Sap treatment. RT-PCR products of a predicted sizes were sequenced directly in both directions for all viruses and viroids, except for *Grapevine rupestris stem pitting-associated virus* (GRSPaV), where RT-PCR products were ligated into the pGEM-Teasy Vector Systems cloning kit (Promega) and then transformed into *Escherichia coli* DH-5α competent cells. Blue/white screening was performed on the LB/carbenicillin/IPTG/X-gal/agar plates. The positive clones were randomly picked and then colony PCR was performed using specific primers (RSP 52/RSP 53) (Supplementary Table 2). After purification, direct RT-PCR or cloned products were sequenced using an Applied Biosystems 3130 Genetic Analyzer. After sequencing, the forward and reverse traces were trimmed and assembled using CodonCode Aligner 9.0.1 (CodonCode Corporation). All sequences generated in this study were deposited in the NCBI GenBank database.^3^ The generated virus and viroid sequences were compared using the ClustalW program (Thompson et al., 1994) implemented in MEGA X software (Kumar et al., 2018). A p-distance model was applied for nucleotide (nt) and deduced amino acid (aa) divergence sequence analysis. Phylogenetic trees were constructed using MEGA X software. The Modeltest implemented in MEGA X was applied to investigate the best-fitting model of nt substitution. The reliability of the obtained trees was evaluated using the bootstrap method based on 1,000 replicates and bootstrap values lower than 50% were omitted.

## Results and Discussion

### Viruses and Viroids Detected by High-Throughput Sequencing of Virus- and Viroid-Derived Small RNAs

sRNA-seq from the pooled grapevine samples yielded 4,206,135–17,668,261 reads. In VirusDetect pipeline 3,643,531–13,905,492 reads per pool were processed (Supplementary Table 1). Additional results of the detection pipeline are presented in Supplementary Table 1. Using the described approach, nine viruses: *Raspberry bushy dwarf virus* (RBDV), *Grapevine Pinot gris virus* (GPGV), *Grapevine rupestris stem pitting-associated virus* (GRSPaV), *Grapevine fanleaf virus* (GFLV), *Grapevine leafroll-associated virus 3* (GLRaV-3), *Grapevine fleck virus* (GFkV), *Grapevine Red Globe virus* (GRGV), *Grapevine rupestris vein feathering virus* (GRVFV), *Grapevine Syrah virus-1* (GSyV-1), and two viroids: *Hop stunt viroid* (HSVd) and *Grapevine yellow speckle viroid- 1* (GYSVd-1) were identified. GRGV, GRVFV, and GSyV-1 were detected for the first time in Slovenia (paper in review).

The highest number of viral entities in a library was eight (in libraries 005 and 010), the lowest number was five (in libraries 007, 009, 014), while the remaining libraries contained six viral entities. The genomes of RBDV and GFLV are bipartite and consist of two single-stranded positive-sense RNAs (RNA1 and RNA2), therefore the consensus length, reference coverage, number of contigs and sequencing depth for both, RNA1 and RNA2 are shown (Table 1). GPGV, HSVd, and GYSVd-1 were detected in all analyzed libraries. RBDV and GLRaV-3 were detected in only one library, 005 (“Laški rizling” variety) and 010 (“Refošk” variety), respectively (Table 1).

### Validation of Predicted Infections by RT-PCR, Direct Sanger Sequencing and Cloning, Sequence Analysis and Phylogenetic Studies

#### Raspberry Bushy Dwarf Virus

The natural occurrence of RBDV in grapevine was first confirmed in Slovenia in “Laški rizling” and “Štajerska belina” using DAS-ELISA and IC-RT-PCR (Mavrič et al., 2003). Reports that this virus infecting grapevines are rare, except in Slovenia it has also been reported in neighboring Sebria and Hungary (Jevremovic and Paunovic, 2011; Pleško et al., 2012; Czotter et al., 2018). In our study, RBDV was found only in “Laški rizling” variety (005 library). It was found in this variety in all Slovenian wine-growing regions (Mavrič Pleško et al., 2009). Complete or almost complete reference coverage of both RNA1 (100%) and RNA2 (98.7%) was obtained (Table 1). All four samples were confirmed positive with RT-PCR and partial RNAs2 were Sanger sequenced and deposited in NCBI (GenBank accession no. OK139039-OK139042). The MP sequences of our isolates shared 100% nt identity (100% aa identity), while in the CP gene region they shared 98.18–99.55% nt identities (97.95–100% aa identities). Considering phylogenetic analysis of partial sequences of the CP gene (438 bp), our isolates clustered among other isolates of *Vitis* sp. retrieved from NCBI and they were clearly separated from isolates of *Rubus* sp. (Supplementary Figure 1), which was also reported in other studies (Mavrič Pleško et al., 2009, 2020; Valasevich et al., 2011).

#### Grapevine Pinot Gris Virus

**FIGURE 1:**
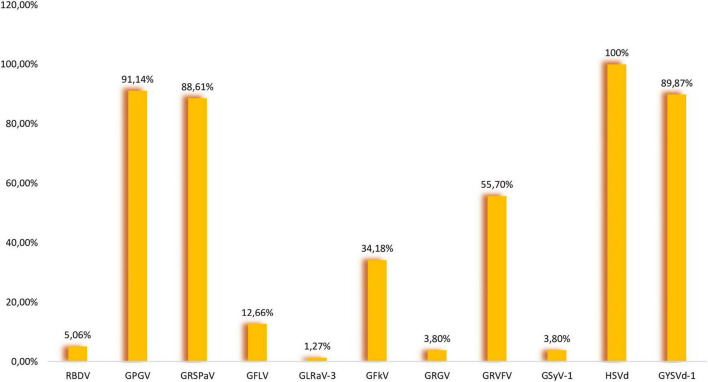
Prevalence of nine viruses and two viroids in analyzed samples.

GPGV is an emerging virus associated with grapevine leaf mottling and deformation (GLMD) disease (Giampetruzzi et al., 2012), but has not yet been included in certification programs in Europe. In Slovenia, the first symptoms (shortened internodes, mottling, deformation, and poor leaf development) were observed in 2001, and samples were tested for eight viruses (ArMV, CLRV, GFLV, RBDV, SLRSV, TBRV, ToRSV, and TRSV), but none was confirmed by DAS-ELISA (Mavrič Pleško et al., 2014). In 2012, GPGV was discovered in Italy using sRNA-seq (Giampetruzzi et al., 2012), and in 2014 its occurrence was reported also from Slovenia (Mavrič Pleško et al., 2014). These authors reported that GPGV seems to be widespread in the Primorska wine-growing region, but they also observed it in different parts of Slovenia. In addition to Italy, the virus seems to be widespread in other neighboring countries, Hungary (17 out of 18 libraries) and Croatia (61.97%) (Czotter et al., 2018; Hančević et al., 2021). Based on HTS data, GPGV was the most prevalent virus in our study. It was found in all 12 libraries (95.3–100% coverage of complete reference sequences) (Table 1). Seventy-two out of 79 samples were positive (91.14%) (Figure 1). Forty sequences were generated and deposited in NCBI (GenBank accession no. OK139043-OK139082). A polymorphism showing C/T variation introducing a premature termination codon was observed in the MP sequence. The C/T polymorphism was observed in 13 samples making MP shorter by 18 nt (6 aa). This polymorphism was also observed for isolates analyzed in some other studies (Reynard, 2015; Saldarelli et al., 2015; Czotter et al., 2018; Abou Kubaa et al., 2020). The MP sequences of 40 Slovenian isolates shared nt identities of 93.94–100% (87.79–100% aa identities). 40 Slovenian CP sequences shared pairwise nt identities of 94.53–100% (97.86–100% aa identities). The phylogenetic tree was constructed using part of the sequences of the MP gene and CP gene (718 nt) and it showed partitioning of our isolates into two clades with isolates from geographically relatively close countries (Supplementary Figure 2).

#### Grapevine Rupestris Stem Pitting-Associated Virus

Using the HTS approach, GRSPaV (member of the rugose wood complex) was detected in 10 libraries (Table 1). In eight libraries complete reference sequences were covered 48.4–99.1%, while in other two libraries, 012 (“Zeleni Sauvignon” variety) and 015 (“Malvazija” variety), only partial genome sequences were covered 62.3 and 81.4%, respectively. In all libraries GRSPaV had a low sequencing depth (5.3X–16.6X). It was confirmed in all libraries where it was predicted with RT-PCR, and even in two other libraries (007 and 014), where it was not detected by sRNA-seq/VirusDetect pipeline. In library 007 (“Rebula” variety) all eight samples were infected, while in library 014 (“Malvazija” variety) only two samples were infected. In Hungary, using sRNA-seq approach, the same contradictory results were reported by three independent studies (Czotter et al., 2018; Demian et al., 2020; Turcsan et al., 2020). The authors indicated that this could be due to technical issues, the possibility that concentrations were under detection threshold due to the bulk sequencing strategy or deeper biological aspects, as a possible long coexistence between grapevine and GRSPaV resulted in mutual adaptation (Gambino et al., 2012), and potentially the plant immune response was not activated. The reason why GRSPaV was not detected by sRNA-seq in some libraries in different studies conducted in different countries requires further studies. Overall, 70 out of 79 samples were tested positive (88.61%), making GRSPaV the second most abundant virus (Figure 1). The selected RT-PCR products were directly sequenced, but due to different genetic variants in the same sample, we were not able to generate high quality sequences, therefore cloning was performed. One RT-PCR product was selected from each variety. Three white colonies were randomly picked from each variety, and after colony PCR, three products were obtained for Laški rizling 3/45B, Rebula 16/1B, Zeleni Sauvignon 14/2P, and Malvazija 20/48P, and one product for Refošk 10/3B and Pokalca 9/27G. In total, 14 products were sequenced (GenBank accession no. OK138921–OK138934), and all were different from each other, even when originating from the same plant. The highest overall mean distance was revealed for three variants of Laški rizling 3/45B (17.14%), and the lowest overall mean distance for Malvazija 20/48P (6.32%). While the overall mean distance for all 14 sequenced variants was 14.06%. It can be concluded that at least three genetic variants exist in the selected samples which differ extensively in the analyzed genome region. The high genetic diversity could be due to the lack of proofreading activity of RdRp, errors in genome replication, frequent recombination, and grafting of individual plants onto differentially infected rootstocks and scions (Glasa et al., 2017). The phylogenetic tree also showed that the genetic variants, even if from the same plant, clustered in different clades (Supplementary Figure 3).

#### Grapevine Fanleaf Virus

GFLV, responsible for a fanleaf degeneration disease and one of the viruses causing the most significant damages on vines (Andret-Link et al., 2004), was detected in two of our libraries, 013 (“Zeleni Sauvignon” variety) and 016 (“Pokalca” variety) (Table 1). RNA1 was covered 92 and 71.7%, respectively, while RNA2 was covered 90.7 and 92.1%, respectively. Validation by RT-PCR using published primers resulted in one sample positive from bulk 013 and three samples from bulk 016. All positive samples were sequenced (GenBank accession no. OK139035–OK139038). Three isolates from “Pokalca” variety shared 99.67 or 99.84% nt identities (99.5 or 100% aa identities), whereas the isolate Zeleni Sauvignon 16/3P differed greatly from the Pokalca isolates with 87.27 or 87.44% nt identities (96.49 or 96.99% aa identities). In addition to the differences between our isolates they also differed from the isolates deposited in NCBI. Pokalca isolates shared the highest identity, 90.62 or 90.79%, with the isolate from France (MG731624), while Zeleni Sauvignon 16/3P shared the highest identity 91.29% with the isolate from Switzerland (MG731616). High sequence variation between GFLV isolates of partial or complete RNA2 (2A^HP^, 2B^MP^, and 2C^CP^) has been reported in several studies (Naraghi-Arani et al., 2001; Fattouch et al., 2005; Pompe-Novak et al., 2007; Elbeaino et al., 2014). This virus does not possess proofreading activity of RdRp and the large genetic variability indicates that the GFLV genome consists of quasispecies populations (Naraghi-Arani et al., 2001). Phylogenetic analysis showed that our isolates differed in the region of partial RNA2 from isolates retrieved from NCBI, even from the previously characterized isolates from Slovenia (Pompe-Novak et al., 2007), but they were the closest to the isolates from Italy and France (Supplementary Figure 4). Due to the differences observed among sequences, we designed new primers based on our sequencing data and repeated analysis. Positive amplifications were observed for additional two samples from bulk 013 and for four samples from bulk 016.

#### Grapevine Leafroll-Associated Virus 3

GLRaV-3 is the major causal agent of one of the most detrimental grapevine diseases named as grapevine leafroll disease (GLD) (Maree et al., 2013). GLRaV-3 was detected only in library 010 (“Refošk” variety). The reference sequence GQ352631 was 98.6% covered and a sequencing depth of 39.6X (Table 1). With the primer pair amplifying the CP gene region, only one sample (Refošk 11/4P) was positive, therefore we used the primer pair amplifying the HSP70h gene region and the same result was obtained. The sequencing of CP gene region of Refošk 11/4P (GenBank accession no. OK138920) showed the highest nt identity (99.76%) with 15 sequences originating from Greece, Portugal, United States, Canada and Pakistan and 3 sequences of unknown origin. Phylogenetic analysis showed that our isolate clustered together with the isolate from Portugal (Supplementary Figure 5). GLRaV-3 was the least prevalent virus in our sample set. Other grapevine leafroll-associated viruses, members of *Ampelovirus* or *Closterovirus* genus, were not detected. The main reason for the lack of detection of leafroll-associated viruses can be explained by the fact that after the mass selection, all selected vines (potential preclonal vines—ELITE) were screened with ELISA tests, which have a fairly good detection for viruses of GLD, therefore at that step, all infected vines were excluded by further selections and propagation. We are aware that serological tests can have quite large deviations in the detection of viruses, but in this case, it seems that we have been quite successful in the leafroll-associated viruses detection with the ELISA test.

#### Grapevine Fleck Virus

GFkV was detected in eight libraries. In five libraries (005, 007, 008, 010, and 012) complete genome reference sequences (AJ309022 or KT000362) were covered with 76.9–82.1%, while in two libraries of “Malvazija” variety (014 and 015), reference sequence AJ309022 was covered only with 30.4 and 32.2%, respectively, and in library 011 partial sequence KF417610 was covered with 33.3%. GFkV was validated in all predicted libraries and for 34 samples we got positive RT-PCR result. All samples were sequenced, but the results showed that seven products belonged to GRVFV, which is consistent with reports from Czotter et al. (2018) from Hungary, indicating high similarity between these two viruses and possible cross-amplification with primers. Sequences of two samples, Laški rizling 3/56B and 3/64B had lower quality, therefore they were excluded from further analysis. Twenty-five GFkV sequences were generated and deposited in NCBI (GenBank accession no. OK139010–OK139034). They shared nt identities of 91.6–100% (93.7–100% aa identities). GFkV is phloem-limited, not mechanically transmissible, and spreads by grafting and infected propagating material (Sabanadzovic et al., 2017). Our isolates shared the highest nt identities with isolates from Bosnia and Herzegovina, North Macedonia, Hungary and the United States. Phylogenetic studies showed that the sequenced isolates clustered in different clades depending on the variety, except “Laški rizling,” with isolates from neighboring countries, while all samples of “Refošk” variety cluster together with isolate from the United States (Supplementary Figure 6). A few decades ago the predecessors of our samples were grafted onto untested rootstocks imported from neighboring countries and from Davis University in California (Hrček, 1977). It seems that with the rootstocks GFkV was imported, but also a lot of grafts produced in Slovenia were exported in neighboring countries, especially in former Yugoslavia.

In addition to GFkV, three fleck-like viruses (GRGV, GRVFV, and GSyV-1) were detected for the first time in Slovenia (paper in review).

#### Hop Stunt Viroid

HSVd has a wide natural host range from different botanical families. Slovenia is one of the major hop producers, and the first report that HSVd infects hops in Slovenia was published in 2012 (Radisek et al., 2012), while it was recently confirmed on grapevines in co-infection with GV-Sat, GLRaV-1, GLRaV-2, GRSPaV, GPGV, and GYSVd-1 (Miljanić et al., 2021). According to the HTS results, HSVd was present in all libraries (Table 1). In ten libraries the reference sequence KJ810551 was covered 100%, while in the other two libraries (008 and 014) the reference sequence KY508372 was covered 99.4% (Table 1). It was validated by RT-PCR and all samples were positive. Forty complete genome sequences were generated and deposited in NCBI (GenBank accession no. OK138935–OK138974). Thirty-eight isolates were 100% identical to each other, while two isolates (Pokalca 3/4P and Pokalca 3/6P) were identical and shared 98% identities with other isolates. In the genome of Pokalca 3/4P and 3/6P isolates, insertions were observed at positions 123 and 257, while SNPs were observed at positions 171, 172, 238, 244, 259, and 260 (Supplementary Figure 9). Also, phylogenetic tree showed that this two isolates clustered completely different from other isolates (Supplementary Figure 7).

#### Grapevine Yellow Speckle Viroid-1

According to our analysis GYSVd-1 was found in all analyzed libraries with 100% references coverage (Table 1). According to RT-PCR, 71 samples were positive (89.87%) (Figure 1). To our knowledge, nine GYSVd-1 sequences of Slovenian autochthonous grapevine varieties have been deposited in NCBI so far (Štajner et al., 2019). In this study, 35 complete genome sequences were generated and deposited in NCBI (GenBank accession no. OK138975–OK139009). GYSVd-1 was less prevalent and showed higher genetic diversity than HSVd. Slovenian GYSVd-1 sequences had 95.35–100% nt identities. Multiple alignment with ClustalW revealed InDel mutations at four positions in the genome (63, 92, 163, and 287) (Supplementary Figure 10). Phylogenetic analysis showed that analyzed GYSVd-1 isolates clustered in different clades regardless of variety (Supplementary Figure 8).

### Co-infections in Analyzed Samples

**FIGURE 2:**
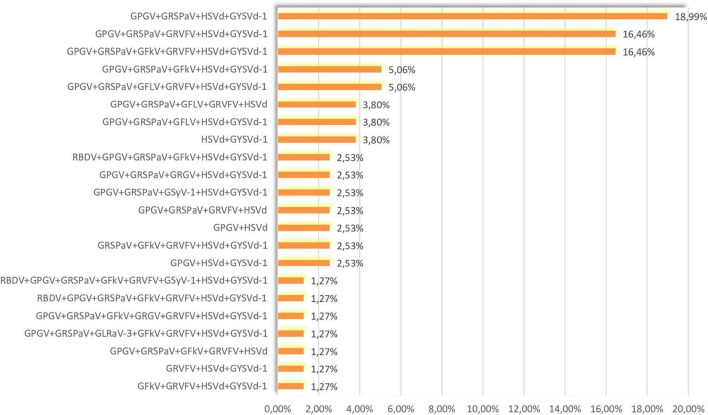
Percentages of co-infections in analyzed samples.

**FIGURE 3:**
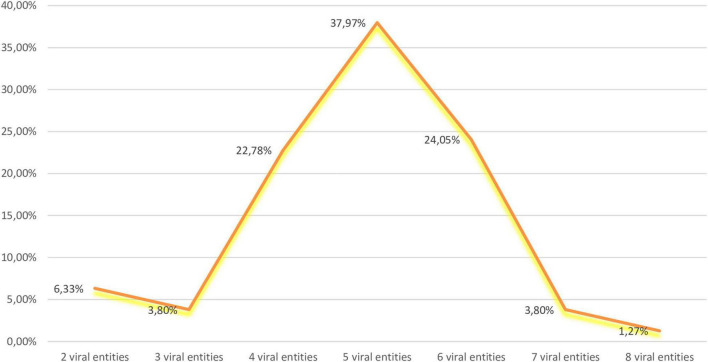
Percentages of mixed infections in analyzed samples.

GPGV, GRSPaV, HSVd, and GYSVd-1 were the most prevalent in our sample set (Figure 1) and their co-infection were the most common (18.99%) (Figure 2). The second most prevalent co-infections were GPGV + GRSPaV + GRVFV + HSVd + GYSVd-1 and GPGV + GRSPaV + GFkV + GRVFV + HSVd + GYSVd-1 (16.46%) (Figure 2). There were no vines that were free of viruses or viroids, and there were no vines that were infected with only one viral entity. The highest number of tested plants were infected with five viral entities (37.97%), followed by six (24.05%) and four viral entities (22.78%), while one sample (Laški rizling 3/45B) was infected with eight viruses/viroids (Figure 3).

## Conclusion

The main advantage of using the HTS approach is the complete insight into virome of the analyzed samples (Czotter et al., 2018). When screening the virome status of selected plants the HTS approach is considered method of choice. The HTS approach used for virome screening is mainly based on bulked samples, which is cost effective, because in the analysis usually a lot of samples are included, and the main limitation is the possibility that due to the bulk sequencing strategy viral concentrations may be under detection threshold in some cases. In our study all individual samples were tested with RT-PCR for each HTS predicted infection, and all obtained results were consistent, except for GRSPaV, but due to the fact that similar results related to inconsistent detection of GRSPaV were obtained in different studies, it may have deeper biological aspect and required further analysis which are discussed.

The present study gives us a detailed insight into the virome status of preclonal candidates of autochthonous and local grapevine varieties in the Primorska wine-growing region of Slovenia. In this study significant number of sequences were generated for different viral pathogens and could further improve their routine diagnostics, which is especially important as they cannot be controlled by conventional plant protection methods.

## Data Availability Statement

The datasets presented in this study can be found in online repositories. The names of the repository/repositories and accession number(s) can be found in the article/Supplementary Material.

## Author Contributions

VM: HTS and bioinformatics analysis, validation of HTS data, data analysis, and writing the original draft. UK: HTS and bioinformatics analysis. DR: provided the plant material, review and editing of the original draft. AŠ: provided the plant material. JJ and NŠ: experimental design, review and editing of the original draft. All authors contributed to the article and approved the submitted version.

## Conflict of Interest

The authors declare that the research was conducted in the absence of any commercial or financial relationships that could be construed as a potential conflict of interest.

## Publisher’s Note

All claims expressed in this article are solely those of the authors and do not necessarily represent those of their affiliated organizations, or those of the publisher, the editors and the reviewers. Any product that may be evaluated in this article, or claim that may be made by its manufacturer, is not guaranteed or endorsed by the publisher.

## Footnotes

1
https://pxweb.stat.si/SiStat/en

2
http://virusdetect.feilab.net

3
www.ncbi.nlm.nih.gov
