# Supplementary material for: Virome Status of Preclonal Candidates of Grapevine Varieties (Vitis vinifera L.) From the Slovenian Wine-Growing Region Primorska as Determined by High-Throughput Sequencing
Source: Front Microbiol. 2022 Feb 21;13:830866. doi: 10.3389/fmicb.2022.830866 (PMC8899541; doi:10.3389/fmicb.2022.830866)
Supplement: Supplementary file 1 [file Data_Sheet_1.docx]

Supplementary Material

**Supplementary Figure 1.** Neighbor-joining method and Kimura-2 parameter model Gamma distributed based on nucleotide sequences of 33 RBDV isolates. Bootstrap analysis were performed with 1000 replicates and only bootstrap values higher than 50% are shown next to relevant branches. Scale bars: substitutions per site. Slovenian sequences generated in this study are shown in blue.

**Supplementary Figure 2.** Maximum Likelihood method and Tamura 3-parameter model with invariable sites based on nucleotide sequences of 64 GPGV isolates. Bootstrap analysis were performed with 1000 replicates and only bootstrap values higher than 50% are shown next to relevant branches. Scale bars: substitutions per site. Slovenian sequences generated in this study are shown in blue.

**Supplementary Figure 3.** Neighbor-joining method and Tamura 3-parameter model Gamma distributed based on nucleotide sequences of 82 GRSPaV isolates. Bootstrap analysis were performed with 1000 replicates and only bootstrap values higher than 50% are shown next to relevant branches. Scale bars: substitutions per site. Slovenian sequences generated in this study are shown in blue. Different genetic variants from the same sample are indicated.

**Supplementary Figure 4.** Neighbor-joining method and Tamura 3-parameter model Gamma distributed based on nucleotide sequences of 42 GFLV isolates. Bootstrap analysis were performed with 1000 replicates and only bootstrap values higher than 50% are shown next to relevant branches. Scale bars: substitutions per site. Slovenian sequences generated in this study are shown in blue.

**Supplementary Figure 5.** Maximum Likelihood method and Kimura-2 parameter model Gamma distributed with invariable sites based on nucleotide sequences of 33 GLRaV-3 isolates. Bootstrap analysis were performed with 1000 replicates and only bootstrap values higher than 50% are shown next to relevant branches. Scale bars: substitutions per site. Slovenian sequence generated in this study is shown in blue.


**Supplementary Figure 6.** Maximum Likelihood method and Hasegawa-Kishino-Yano model Gamma distributed based on nucleotide sequences of 43 GFkV isolates. Bootstrap analysis were performed with 1000 replicates and only bootstrap values higher than 50% are shown next to relevant branches. Scale bars: substitutions per site. Slovenian sequences generated in this study are shown in blue.

**Supplementary Figure 7.** Neighbor-joining method and Jukes-Cantor model Gamma distributed based on nucleotide sequences of 69 HSVd isolates. Bootstrap analysis were performed with 1000 replicates and only bootstrap values higher than 50% are shown next to relevant branches. Scale bars: substitutions per site. Slovenian sequences generated in this study are shown in blue.

**Supplementary Figure 8.** Neighbor-joining method and and Jukes-Cantor model Gamma distributed based on nucleotide sequences of 63 GYSVd-1 isolates. Bootstrap analysis were performed with 1000 replicates and only bootstrap values higher than 50% are shown next to relevant branches. Scale bars: substitutions per site. Slovenian sequences generated in this study are shown in blue.


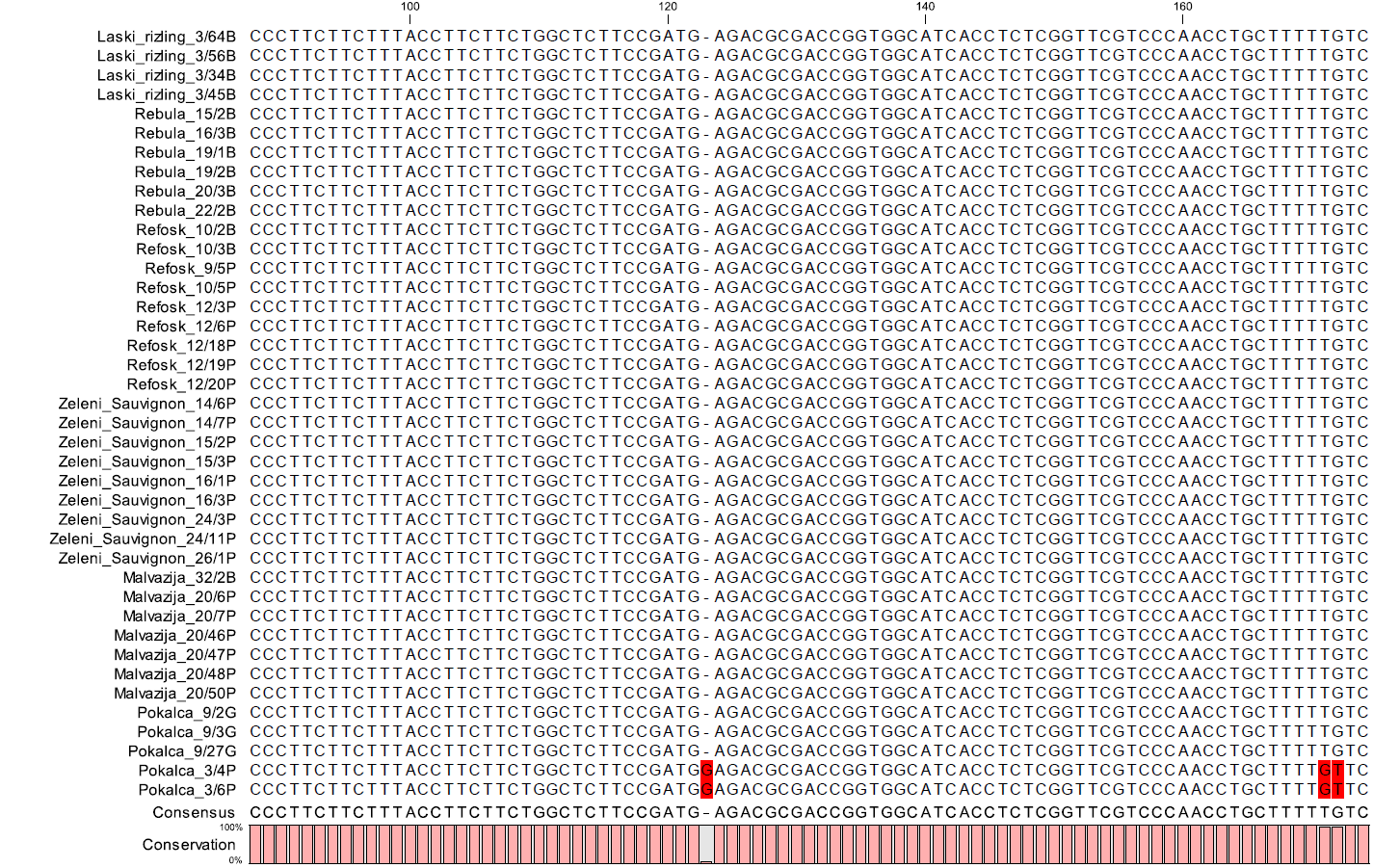


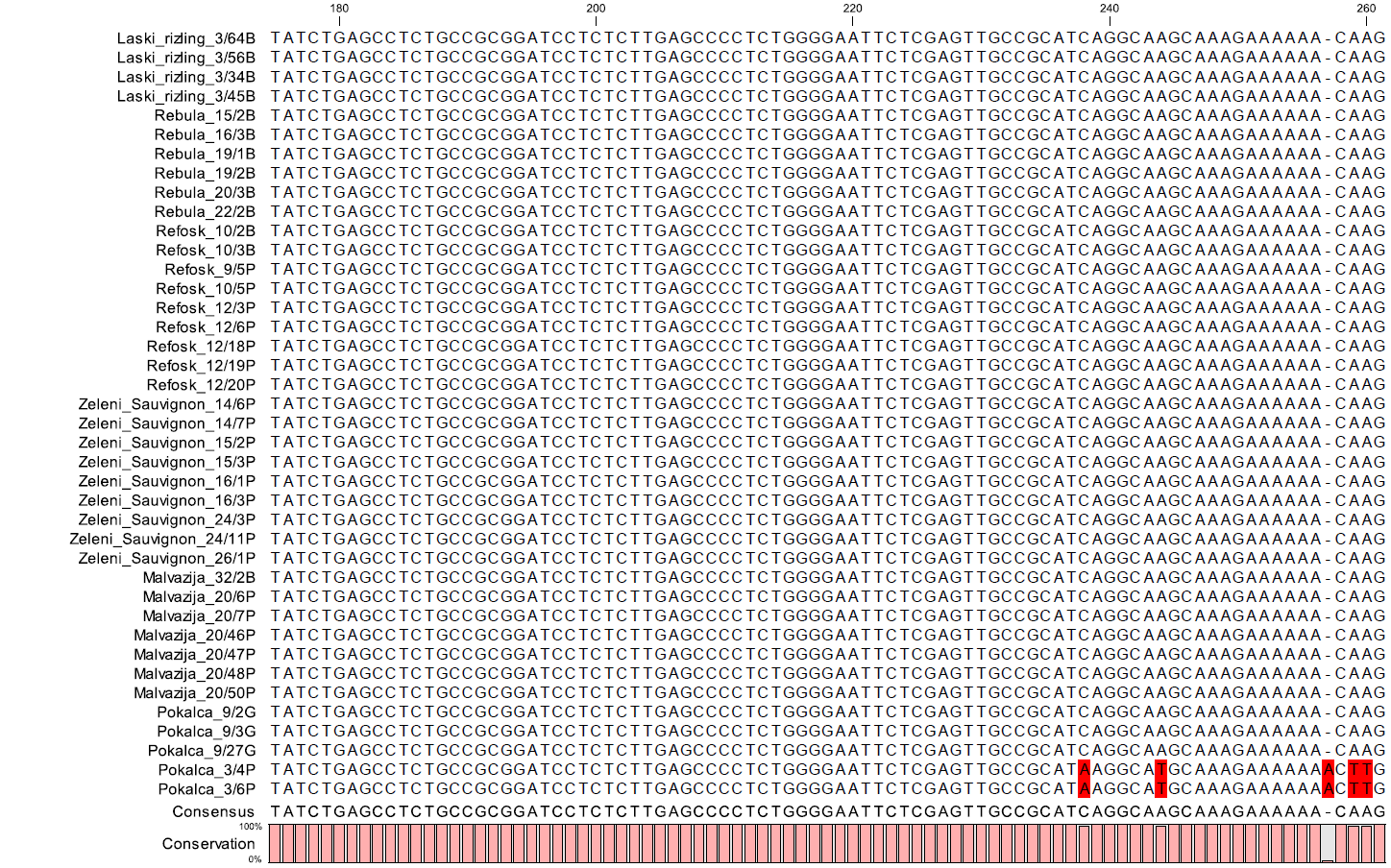


**Supplementary Figure 9.** Nucleotide sequence alignment (CLC Genomic Workbench, ver 21.0.5) between 88 and 261 nt positions of 40 Slovenian HSVd sequences. In the genome of Pokalca 3/4P and 3/6P isolates, insertions were observed at positions 123 and 257, while SNPs were observed at positions 171, 172, 238, 244, 259 and 260.


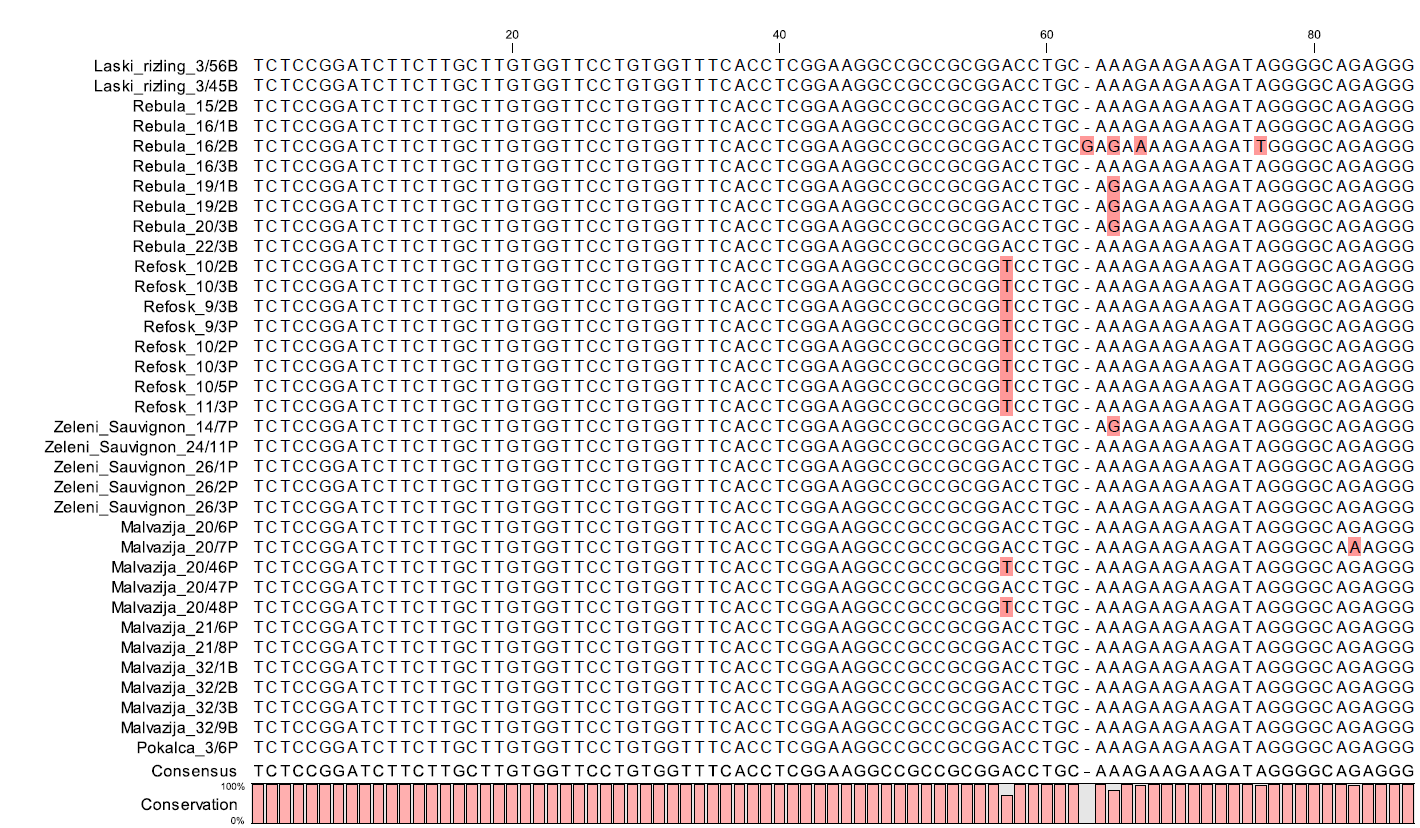


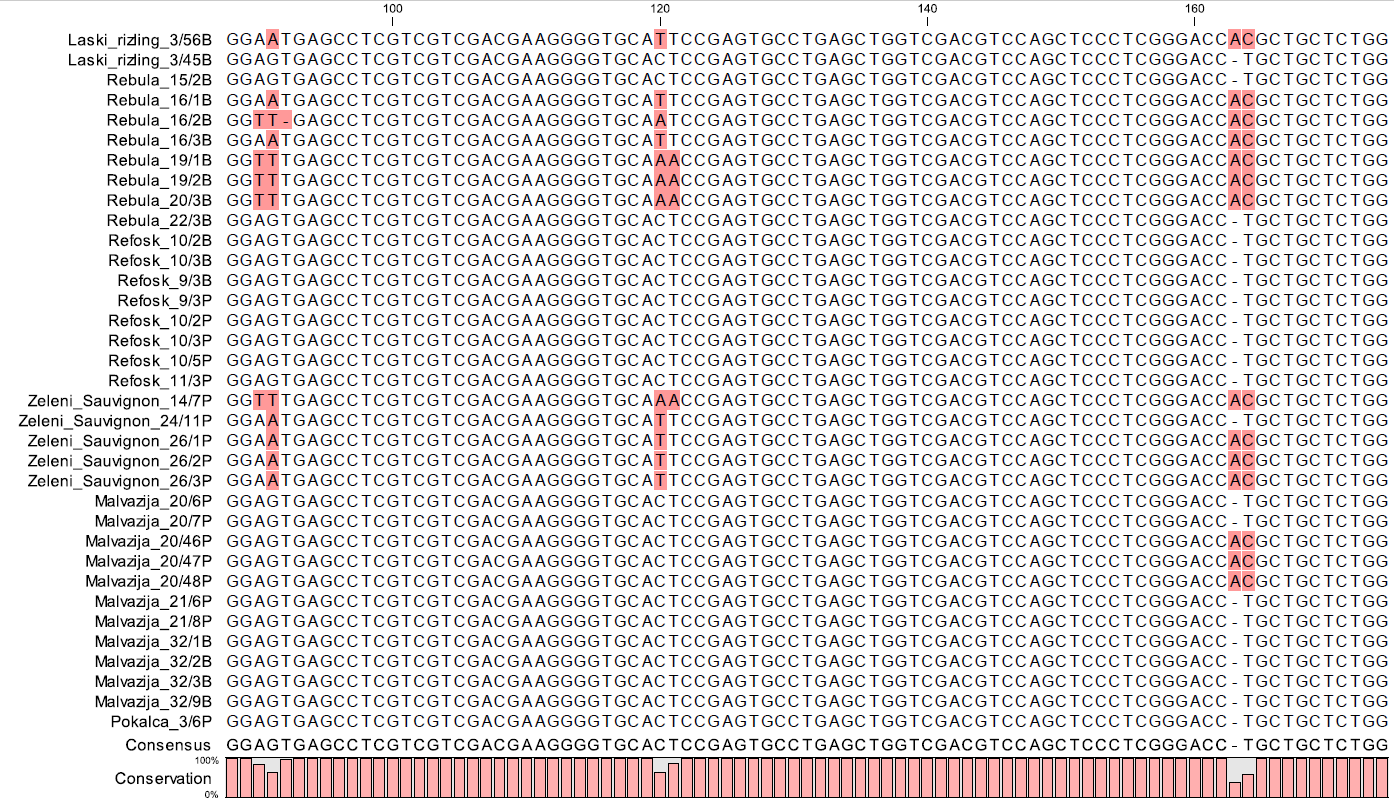


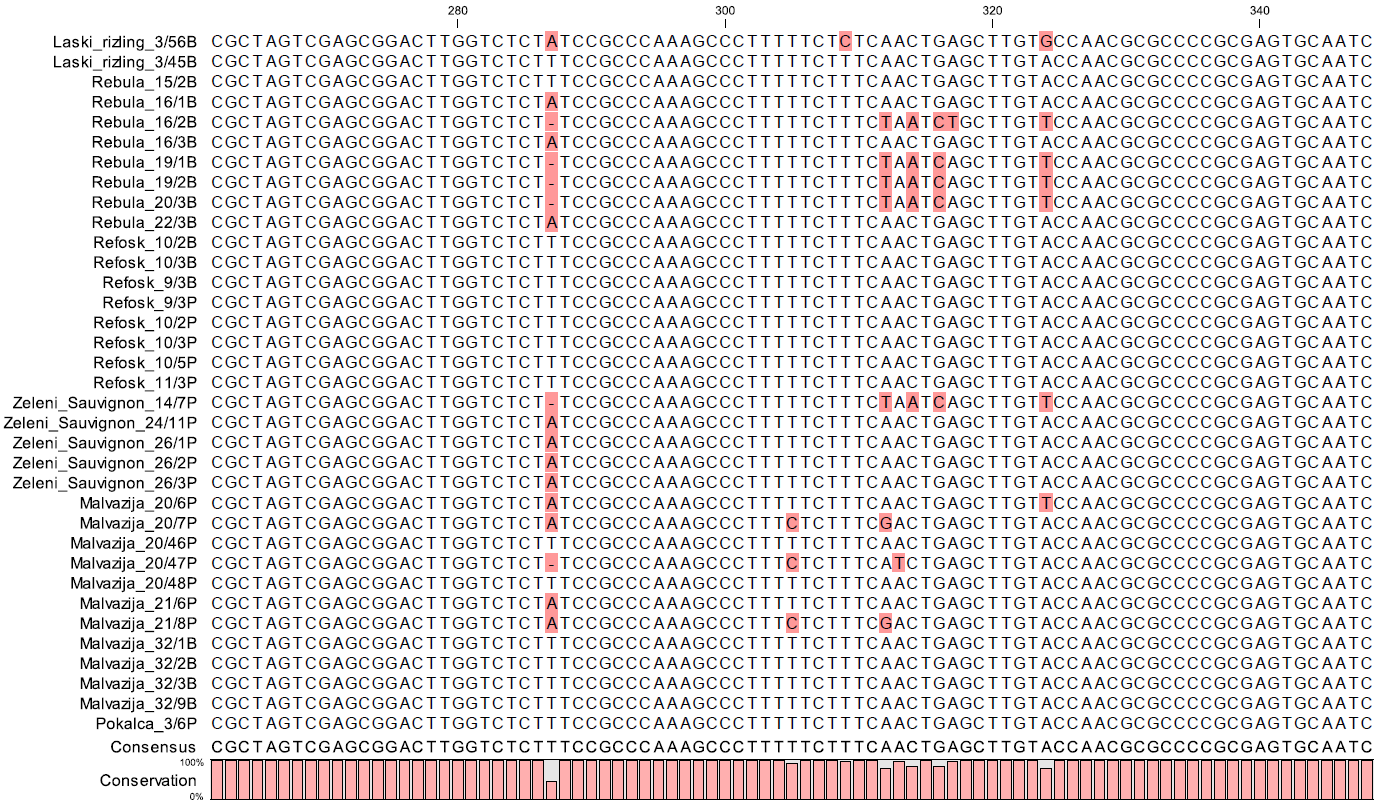


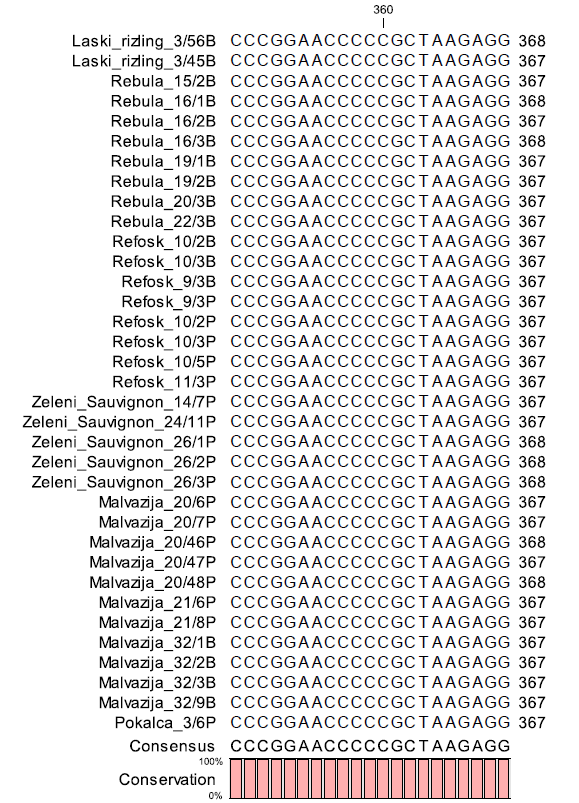


**Supplementary Figure 10.** Nucleotide sequence alignment (CLC Genomic Workbench, ver 21.0.5) of 35 Slovenian GYSVd-1 complete genome sequences. InDel mutations were observed at four positions in the genome (63, 92, 163 and 287).
